# Manipulation of Displaced Distal Radial Fractures in the Superelderly: Prediction of Malunion and the Degree of Radiographic Improvement

**DOI:** 10.1155/2014/785473

**Published:** 2014-10-08

**Authors:** N. D. Clement, A. D. Duckworth, C. M. Court-Brown, M. M. McQueen

**Affiliations:** Department of Orthopaedics and Trauma, The Royal Infirmary of Edinburgh, Little France, Edinburgh EH16 4SA, UK

## Abstract

Superelderly patients (≥80 years old) account for 20% of all distal radial fractures and are at an increased risk of malunion. The primary aim of this study was to identify predictors of malunion and the degree of improvement in the fracture position offered by closed manipulation of displaced distal radial fractures in the superelderly. We retrospectively identified 228 displaced distal radial fractures in superelderly patients from a prospective database of 4024 distal radial fractures. The inclusion criterion was a patient that underwent closed manipulation as their primary intervention. The majority of patients (n = 196, 86%) were defined as having a malunion. A premanipulation dorsal angulation of greater than 25 degrees (*P* = 0.047) and an ulnar variance of 6 mm or more (P = 0.02) significantly increased the risk of malunion. The premanipulation dorsal angulation was a significant independent predictor of the degree of improvement in the final dorsal angulation (P < 0.001) and ulnar variance (P = 0.01). Patients with a high risk of malunion or poor improvement in the fracture position can be identified before manipulation and these patients may benefit from primary surgical intervention.

## 1. Introduction

Fractures of the distal radius account for 16% of all fractures and are the most prevalent fracture that orthopaedic surgeons have to manage [1]. Stable fractures can be managed conservatively with the expectation of a good functional outcome [2, 3]. The management of unstable fractures of the distal radius in the elderly remains controversial [4]. The functional outcome of displaced fractures is generally accepted to correlate with the anatomical reduction of the fracture [2, 3, 5].

It is predicted that there will be an increase in the elderly population over the next decade in the United Kingdom (UK), and currently the fastest growing population is the “oldest old” [6]. It is anticipated that by 2030 the population age of 80 years or more will have doubled [7]. The term “superelderly” has been used in orthopaedics to describe those patients greater than 80 years of age [8, 9]. Superelderly patients account for approximately 20% of all distal radial fractures [10], which will probably increase in the future due to their growing population and will form a greater proportion of the orthopaedic workload.

The primary management of displaced distal radial fractures is close manipulation [11]. However, increasing age, fracture comminution, and dependency are predictors of fracture stability and, if present, are more likely to lead to malunion [12]. These predictors are more likely to be present in the superelderly population and hence are more likely to fail nonoperative management by close manipulation alone. If the risk of malunion and degree of improvement offered by closed manipulation of the fracture could be calculated prior to manipulation, superelderly patients with a high risk of malunion could be identified. These patients may benefit from early operative intervention, avoiding delay and the inconvenience of a failed manipulation.

The primary aim of this study was to identify predictors of malunion and degree of improvement in the fracture position offered by closed manipulation of displaced distal radial fractures in the superelderly. The secondary aim was to describe the epidemiology of superelderly patients with displaced distal radial fractures.

## 2. Patients and Methods

### 2.1. Demographic Data

During a 67-month period 28,376 acute fractures were managed at the study centre, of which distal radial fractures accounted for 4024 (14.2%). A distal radial fracture database was prospectively complied, recording demographics, radiographic data, management, and outcome. The mean age for all patients was 59 (14 to 100) years. There were 574 patients aged 80 years or older identified retrospectively who had sustained a distal radial fracture during the study period, of which 228 were displaced and underwent closed manipulation as their primary intervention. There were 213 (93.4%) females and 15 (6.6%) males with a mean age of 83.7 (80 to 98) years. Two patients sustained bilateral fractures.

### 2.2. Database Construction

Fracture management followed a standard protocol. The emergency-room staff undertook the initial assessment and treatment. Fractures deemed to be in an acceptable position were managed with a dorsal plaster slab. If the fracture position was thought to be unacceptable, the emergency-room staff, prior to application of a dorsal plaster slab, performed closed reduction using intravenous regional anaesthesia. The patients were evaluated clinically and radiographically at one week and six weeks after the injury as per the protocol of the study unit.

At approximately one week following the injury, the patients were reviewed by the senior author (M. M. McQueen) in a dedicated research clinic. The clinical, demographic, and radiographic data were recorded and entered into a database either by the senior author (M. M. McQueen) or a research nurse. The premorbid normal level of function of the patient was categorised as independent if they were able to go shopping without assistance or dependent if assistance was needed. The dorsal slab was completed to a below-the-elbow forearm cast with the wrist in slight flexion and ulnar deviation at one week. Patients with a fracture that was displaced were admitted to the orthopaedic trauma unit for further intervention, unless the patient had low functional demands and operative intervention was deemed inappropriate.

### 2.3. Radiographic Measurement Techniques

**Figure 1:**
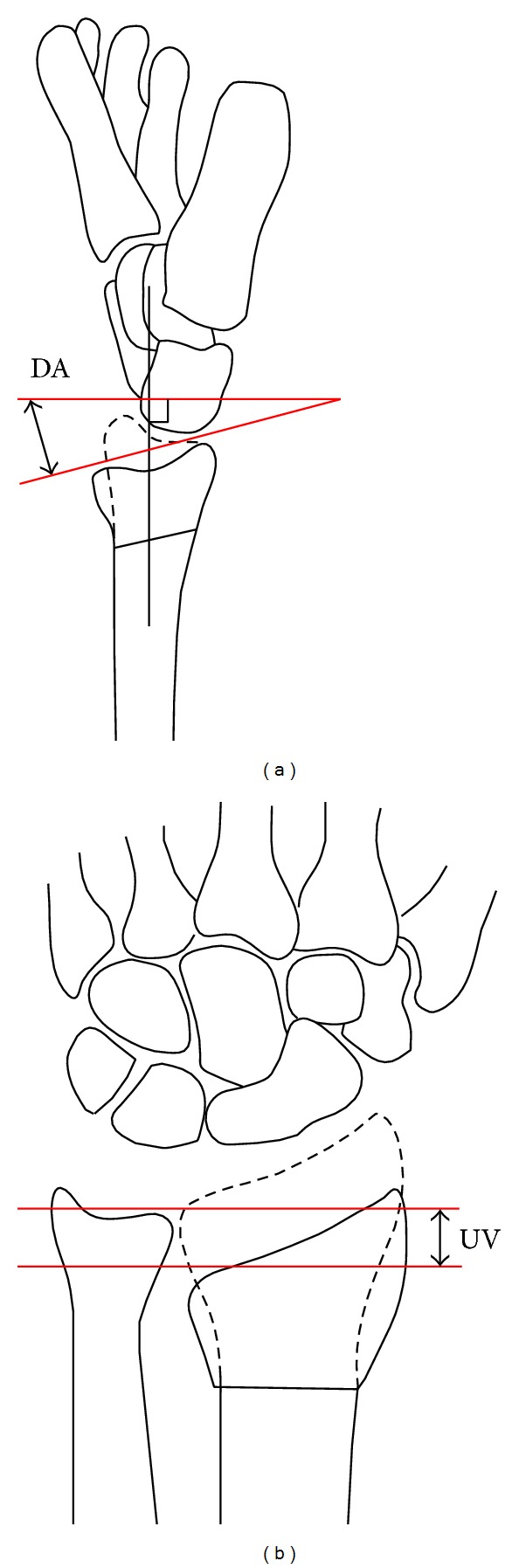
The measurement of dorsal angle (DA) and ulnar variance (UV). These measurements were expressed as a negative for volar angulation and a positive for DA and a positive value for UV if there was radial shortening.

All radiographs (presentation, time of reduction, one week, and six weeks) were measured manually with use of a protractor and a ruler to provide values for the dorsal angle and ulnar variance (Figure 1). The fractures were classified using AO/OTA classification [13]. Metaphyseal comminution was recorded, according to the location, as absent or as involving the dorsal metaphysis, volar metaphysis, or both the dorsal and the volar metaphysis. The senior author (M. M. McQueen) alone was responsible for fracture classification and the assessment of comminution. Adequate reduction was defined as dorsal angle of ≤0 degrees and/or ≤3 mm of radial shortening. Malunion was defined as a dorsal angle of >10 degrees and/or >3 mm of radial shortening.

### 2.4. Statistical Analysis

SPSS version 16.0 software was used for statistical analysis (SPSS Inc., Chicago, IL, USA). Paired and unpaired* t*-tests were used to compare the improvement in the 6-week dorsal angulation, ulna variance relative to premanipulation dorsal angulation and ulna variance, and the effect of case-mix variables upon the improvement in dorsal angulation and ulna variance, respectively. Pearson's correlation coefficient was used to assess the significance of age and premanipulation dorsal angulation and ulna variance upon the 6-week dorsal angulation and ulna variance. Multiple linear (stepwise methodology) and logistic (forward conditional methodology) regression analyses were used to identify significant independent predictors of improvement in dorsal angulation and ulnar variance and risk of malunion. A *P* value of ≤0.05 determined statistical significance.

## 3. Results

The majority of patients were female and were independent (Table 1). The mean age of males was 83.2 (range 80 to 88) years and that of females was 83.7 (range 80 to 98) years (unpaired* t*-test *P* = 0.58). Thirty-seven (16%) of patients sustained an associated fracture during the same injury, of which the commonest mode was a simple fall from standing height (Table 1). Dorsal comminution was observed in most patients, and only one patient sustained a partial articular fracture (Table 1). The mean premanipulation dorsal angulation was 25.2 degrees (10 to 56 degrees, SD 14.9) and radial shortening was 2.9 mm (−4 to 16 degrees, SD 3.1).

An adequate reduction of the distal radial fracture was achieved in 87 patients (38%). Overall there was a significant improvement in the position of the fracture, in both the degree of dorsal angulation and the degree of ulnar variance (Table 2). The position of the fracture 6 weeks after injury had deteriorated, resulting in 196 (86%) patients being defined as having a malunion (Table 2).

**Figure 2:**
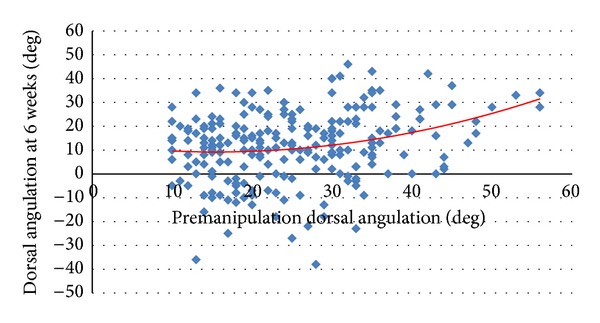
Correlation between premanipulation dorsal angulation and dorsal angulation at 6 weeks.

**Figure 3:**
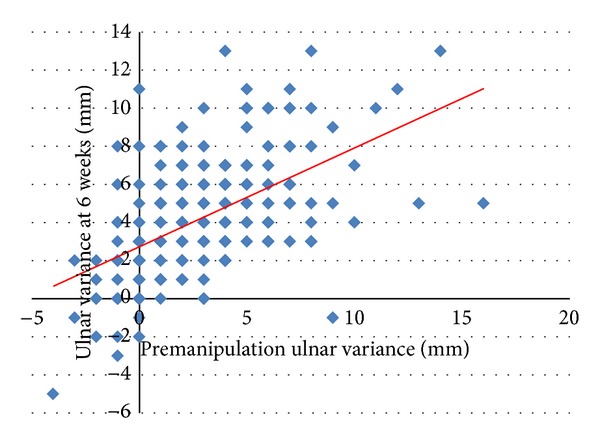
Correlation between premanipulation ulnar variance and ulnar variance at 6 weeks.

There was a significant correlation between premanipulation dorsal angulation (*r* = 0.24 and Pearson correlation *P* < 0.001) and ulnar variance (*r* = 0.54, Pearson correlation *P* < 0.001) with that measured at 6 weeks after injury (Figures 2 and 3). Using these correlations on average a mean dorsal angulation of greater than 25 degrees, or an ulnar variance of 6 mm or more, would result in a malunion at 6 weeks (Figures 2 and 3). Using these values as predictors, a dorsal angulation of greater than 25 degrees (odds ratio (OR) 2.3 and chi-square *P* = 0.047) and an ulnar variance of 6 mm or more (OR 1.2 and chi-square *P* = 0.02) both significantly increased the risk of malunion. These two risk factors were additive (OR 2.7 and chi-square *P* = 0.014), and combining these two predictors alone would result in a test that is 52% sensitive and 72% specific for predicting malunion. This test was a significant independent predictor of malunion when adjusting for other confounding variables (*R* ^2^ = 0.05, OR 2.72, using bivariate regression analysis *P* = 0.017). No other variables were significant and they were not included within the model.

Dorsal comminution, the premanipulation dorsal angulation, and ulnar variance were significant predictors of the degree of improvement in the 6-week dorsal angulation and ulnar variance (Table 3). Premanipulation dorsal angulation was the only significant isolated predictor, after adjusting for other confounding variables, of improvement in the degree of dorsal angulation at 6 weeks (Table 4). Premanipulation dorsal angulation and ulnar variance and dorsal comminution were all significant isolated predictors, after adjusting for other confounding variables, of improvement in the ulnar variance at 6 weeks (Table 4).

## 4. Discussion

We have demonstrated that the majority of displaced distal radial fractures in superelderly patients occur in females, after a simple fall, of which approximately half are functionally independent. Closed manipulation of the distal radial fracture achieved an adequate reduction in one-third of patients, and the majority of patients had an improvement of their fracture position. The degrees of dorsal angulation, ulna variance, and dorsal comminution were independent predictors of improvement in the position of the fracture; however, most patients (86%) went onto a malunion. Those patients with a dorsal angulation of greater than 25 degrees and an ulna variance of more than 6 mm at the time of their injury were at the greatest risk of malunion.

Approximately 30% of distal radial fractures occur in men, and 70% are due to falls from standing height [10]. The prevalence of superelderly male patients sustaining displaced distal radial fractures is less, accounting for only 6%, and 98% are due to falls from standing. This probably relates to the increased fragility of the superelderly population, with a greater prevalence of osteoporosis in female patients [14], resulting in more low energy fractures in women. The incidence of distal radial fractures increases with age, with a peak of 1107/10^5^/year in superelderly women [15]. If the predicted rise in the population is correct this will result in 44 thousand (population estimate of 6 million [7] with an approximate incidence of 730/10^5^/year [10]) distal radial fractures in superelderly patients presenting to orthopaedic trauma services per year in the UK by the year 2030. Assuming 40% will have displaced fractures, as we have demonstrated, this would mean 18 thousand superelderly patients may undergo manipulation. This will have major repercussions upon the orthopaedic trauma workload, and hence efficient and appropriate management of these fragility fractures will be of paramount importance in optimising the care of these patients.

A unique aspect of this study was to define specific cutoff values for dorsal angulation and ulnar variants as independent predictors of malunion of distal radius fractures in the superelderly. Previous studies identifying risk factors for malunion found dorsal comminution to be an independent predictor of malunion [12, 16]. We have also demonstrated that the combination of dorsal angulation of greater than 25 degrees and/or ulnar variance of greater than 6 mm resulted in the greatest risk of malunion in the superelderly patients. The clinical relevance of this is not clear in this low demand group [4]. There is a body of evidence which demonstrates that the functional outcome of distal radial fractures correlates with the end anatomic result [2, 3, 5]. These studies are, however, heterogeneous including patients with a wide age range and include both extra- and intra-articular fracture patterns. Studies focusing on elderly patients and those with low functional demand have shown that malunion does not correlate with an inferior outcome [4]. More recently Grewal and MacDermid [17] demonstrated that for independent elderly patients with displaced extra-articular distal radius fractures, malunion did not result in a diminished functional outcome.

If a malunion does not affect functional outcome then the question could be asked whether it is worth manipulating displaced distal radius fractures in superelderly patients. Beumer and McQueen [18] suggested that for functionally active elderly patients it would be reasonable to assume that functional outcome does correlate with reduction of the fracture, as demonstrated for younger patients. It would also seem reasonable to assume that the degree of malunion may correlate with functional outcome in the elderly. Hence, manipulation of the fracture with improvement in the position, despite being defined as a malunion, may result in an improved outcome. Kelly et al. [19] demonstrated that elderly patients with more than 30 degrees of dorsal angulation and 5 mm of ulnar variance resulted in a poorer outcome. Using our prediction models for improvement in dorsal angulation and ulnar variance for superelderly patients it would be mathematically possible to calculate the improvement potentially offered by manipulation of displaced fractures, for example:

(1) Improvement  in  dorsal  angulation  (DA) =(premanipulation  DA×0.655)−3.174  (constant)Improvement  in  ulna  variance  (UV) =(pre-manipulation  UV×0.534)  +(pre-manipulation  DA×0.032)  +1.433  if  dorsal  comminution−1.617 (constant).

This would enable a patient's final position to be calculated, identifying those patients who would achieve or fail to achieve the radiographic criteria defined by Kelly et al. [19]. Independent superelderly patients who are predicted to fail to achieve these radiographic criteria may benefit from primary surgical intervention, to avoid a wasted intervention and delay in definitive management.

Superelderly patients with a displaced distal radial fracture managed with closed manipulation alone will result in a malunion for the majority of patients. The position of the fracture will, however, be improved by closed manipulation, but whether this improvement results in an enhanced functional outcome is not known. It would seem reasonable to assume that the improvement in the radiographic position of the fracture correlates with a superior functional outcome. This assumption needs to be confirmed to ensure primary manipulation is of functional benefit to superelderly patients and not a waste of healthcare resources and an inconvenience to our patients.

## Figures and Tables

**Table 1:** Case-mix variables for the study cohort.

Case-mix variables	*n* = (%)
Gender	
Male	15 (6.6)
Female	213 (93.4)
Dominant limb	
Yes	93 (40.8)
No	135 (59.2)
Independent	
Yes	126 (55.3)
No	102 (44.7)
Injury mechanism	
Simple fall	224 (98.2)
Fall from height	1 (0.4)
RTA	2 (0.9)
Assault	1 (0.9)
Associated fracture	
Yes	37 (16.2)
No	191 (83.8)
AO Classification	
A	123 (53.9)
B	1 (0.4)
C	104 (45.6)
Dorsal comminution	
Yes	212 (93.0)
No	16 (7.0)

**Table 2:** Dorsal angulation and ulnar variance pre- and postmanipulation and the statistical significance of improvement relative premanipulation measurement.

Time point		Dorsal angulation in degrees (SD)	*P* value∗	Ulnar variance in mm (SD)	*P* value∗
Premanipulation		25.2 (10.1)	—	2.9 (3.1)	—

One week	Absolute	1.2 (9.4)	<0.001	0.9 (2.6)	<0.001
	Improvement	24.0		2.0	

6 weeks	Absolute	11.9 (14.5)	<0.001	4.3 (3.0)	<0.001
	Improvement	13.3		−1.4	

*Paired *t*-test.

**Table 3:** Predictors of improvement in the dorsal angulation and ulna variance at 6 weeks.

Case-mix variables		Improvement in dorsal angulation (degrees)	*r* =	*P* value	Improvement in ulnar variance (mm)	*r* =	*P* value
Gender	Male	13.7	—	0.92∗	1.7	—	0.51∗
	Female	13.3	—		2.1	—	

Age		—	0.05	0.46^†^	—	0.05	0.47^†^

Independent	Yes	14.2	—	0.35∗	1.9	—	0.24∗
	No	12.3	—		2.3	—	

Dorsal comminution	Yes	13.4	—	0.7∗	2.2	—	0.001∗
	No	11.9	—		0.1	—	

AO classification	A	14.0	—	0.15∗∗	2.0	—	0.7∗∗
	B	16.0	—		4.0	—	
	C	12.8	—		2.2	—	

Premanipulation	Dorsal angulation	—	0.43	<0.001^†^	—	0.34	<0.001^†^
	Ulnar variance	—	0.15	0.021^†^	—	0.70	<0.001^†^

*Unpaired *t*-test.

∗∗ANOVA.

^†^Pearson correlation.

**Table 4:** Significant predictors of improvement in dorsal angulation and ulna variance at 6 weeks.

Outcome variable	Risk factor	*B*	95% confidence interval	*P* value∗
Improvement in dorsal angulation (*R* ^2^ = 0.20)	Premanipulation dorsal angulation	0.66	0.47 to 0.84	<0.001
	(constant)	−3.17	−8.14 to −1.79	<0.001

Improvement in ulnar variance (*R* ^2^ = 0.31)	Premanipulation dorsal angulation	0.03	0.01 to 0.06	0.01
	Premanipulation ulnar variance	0.53	0.46 to 0.61	<0.001
	Dorsal comminution	1.43	0.52 to 2.34	0.002
	(constant)	−1.62	−1.88 to −1.35	0.002

*Linearregression analysis.
